# Novel Synthesis of Core-Shell Silica Nanoparticles for the Capture of Low Molecular Weight Proteins and Peptides

**DOI:** 10.3390/molecules22101712

**Published:** 2017-10-12

**Authors:** Sergio G. Hernandez-Leon, Jose Andre-i Sarabia-Sainz, Gabriela Ramos-Clamont Montfort, Ana M. Guzman-Partida, Maria del Refugio Robles-Burgueño, Luz Vazquez-Moreno

**Affiliations:** 1 Centro de Investigación en Alimentación y Desarrollo A.C. Carretera a la Victoria km 0.6 C.P. 83304, Hermosillo 83304, Sonora, Mexico; gerardo.hernandez@estudiantes.ciad.mx (S.G.H.-L.); gramos@ciad.mx (G.R.-C.M.); gupa@ciad.mx (A.M.G.-P.); cuquis@ciad.mx (M.d.R.R.-B.); 2 Universidad de Sonora. Blvd. Luis Encinas y Rosales S/N, Col. Centro, Hermosillo 83000, Sonora, Mexico; andreisarabia@gmail.com

**Keywords:** functionalized core-shell silica nanoparticles, Cibacron Blue, low molecular weight proteins/peptides, high molecular weight proteins, hydrophobic

## Abstract

Silica nanoparticles were functionalized with immobilized molecular bait, Cibacron Blue, and a porous polymeric bis-acrylamide shell. These nanoparticles represent a new alternative to capture low molecular weight (LMW) proteins/peptides, that might be potential biomarkers. Functionalized core-shell silica nanoparticles (FCSNP) presented a size distribution of 243.9 ± 11.6 nm and an estimated surface charge of −38.1 ± 0.9 mV. The successful attachment of compounds at every stage of synthesis was evidenced by ATR-FTIR. The capture of model peptides was determined by mass spectrometry, indicating that only the peptide with a long sequence of hydrophobic amino acids (alpha zein 34-mer) interacted with the molecular bait. FCSNP excluded the high molecular weight protein (HMW), BSA, and captured LMW proteins (myoglobin and aprotinin), as evidenced by SDS-PAGE. Functionalization of nanoparticles with Cibacron Blue was crucial to capture these molecules. FCSNP were stable after twelve months of storage and maintained a capacity of 3.1–3.4 µg/mg.

## 1. Introduction

The identification of LMW proteins in biological fluids is a pivotal need in biomedical research since they might represent a new source of biomarkers predictive of early-stage diseases [1,2,3]. However, the isolation and purification of LMW proteins, mainly from urine [4,5] and blood, is complicated because concentrations are frequently low, under the detection limits for mass spectrometry and conventional immunoassays [2,6,7]. In addition, these proteins/peptides could be subject to rapid degradation by proteases, as well as masked by the presence of more abundant proteins, such as albumin and immunoglobulins, that represent over 90% of plasma proteins [2,6,7]. Therefore, techniques that allow the simultaneous exclusion of high abundance proteins and adsorption of low abundance proteins are needed. In this sense, strategies based on nanotechnology might represent a new alternative to overcome these limitations.

Several types of nanoparticles, such as quantum dots [8,9], silver and gold nanoparticles [10], and hydrogels [11] have been used in applications for diagnosing, monitoring, and treating different diseases. However, these nanoparticles are generally addressed to drug delivery and not to the sequestering or capturing of proteins and peptides. In this matter, Tamburro et al. [12] developed multifunctional hydrogel core-shell nanoparticles functionalized with different molecular baits, including Cibacron Blue, to capture and concentrate biomarkers in biological fluids. However, the synthesis of these particles is laborious and the size obtained is 500–700 nm. Thus, the inherent characteristic properties of a nanoscale particle, mainly the surface area [13], might not be optimally exploited. Consequently, alternative materials for the synthesis of core-shell nanoparticles are needed. In this context, silica is a widely-used compound due to its high stability, particularly because it can be chemically modified with several compounds [14,15,16]. The aim of this work was to synthesize functionalized core-shell silica nanoparticles with the pseudo-affinity molecular bait, Cibacron Blue (core), and a polymeric bis-acrylamide shell, thereby forming a molecular exclusion sieve. Thus, the polymeric shell hinders the entrance of HMW proteins, such as albumin, but allows LMW proteins/peptides to enter and interact with the immobilized Cibacron Blue.

## 2. Results and Discussion

### 2.1. Synthesis of FCSNP and Characterization

**Figure 1:**
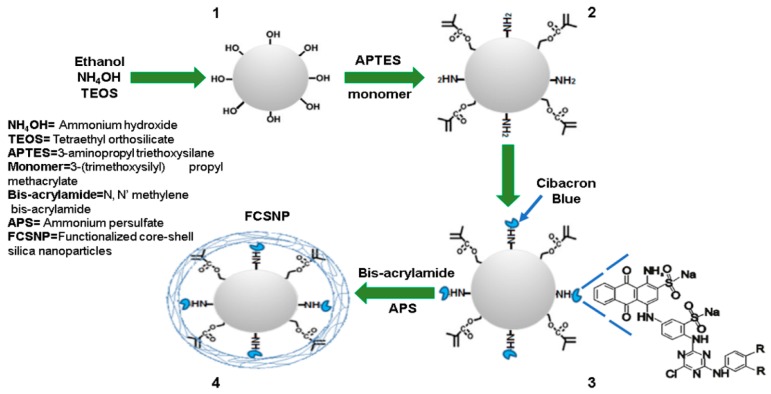
Schematic illustration of the strategy used for the synthesis of functionalized core-shell silica nanoparticles (FCSNP).

A synthesis scheme for FCSNP is illustrated in Figure 1. Functionalization with randomly distributed chemical groups for the addition of molecular bait was achieved by nucleophilic substitution (core) and the addition of an exclusion polymer (shell) by free radical polymerization. Each stage of synthesis was characterized by estimating the particle size and surface charge by dynamic light scattering (DLS) and zeta potential, respectively (Table 1).

The average size of silica nanoparticles (Stage 1) was within the expected size range (50–900 nm) reported by Stöber [16,17]. An increase in the nanoparticle size was obtained after the addition of APTES and the monomer 3-(Trimethoxysilyl) propyl methacrylate (stage 2). These reagents provide amino and ester functional groups, respectively, on the silica nanoparticles surface (core), which are necessary for the chemical modifications that followed. The organic compounds attached to the surface of the nanoparticles form a dense layer that increases the electrostatic repulsion at the surface, increasing the particle size [18]. In this matter, Ferreira et al., 2015 reported an increase of ~70 nm after the modification of silica nanoparticles with APTES. This coincides with our results, since we obtained an increase of ~80 nm after the addition of both APTES and the monomer.

On the other hand, no change in the average size was obtained when Cibacron Blue was added (stage 3). This could be attributed to the hydrophobic nature of the molecule [12,19]. Finally, when the exclusion polymer shell was formed (stage 4), the nanoparticle size increased due to the growth of the polymer chains [20,21], thus forming the molecular sieve on the nanoparticle surface. In this grafting-from approach, crosslinked chains can be grown directly onto the particles to form the shell [21], simply by the addition of a bi- or multifunctional crosslinker as a comonomer in the polymerization. The changes in the surface charge (zeta potential) after every functionalization stage (Table 1) confirm the effective addition of each component [22].

**Figure 2:**
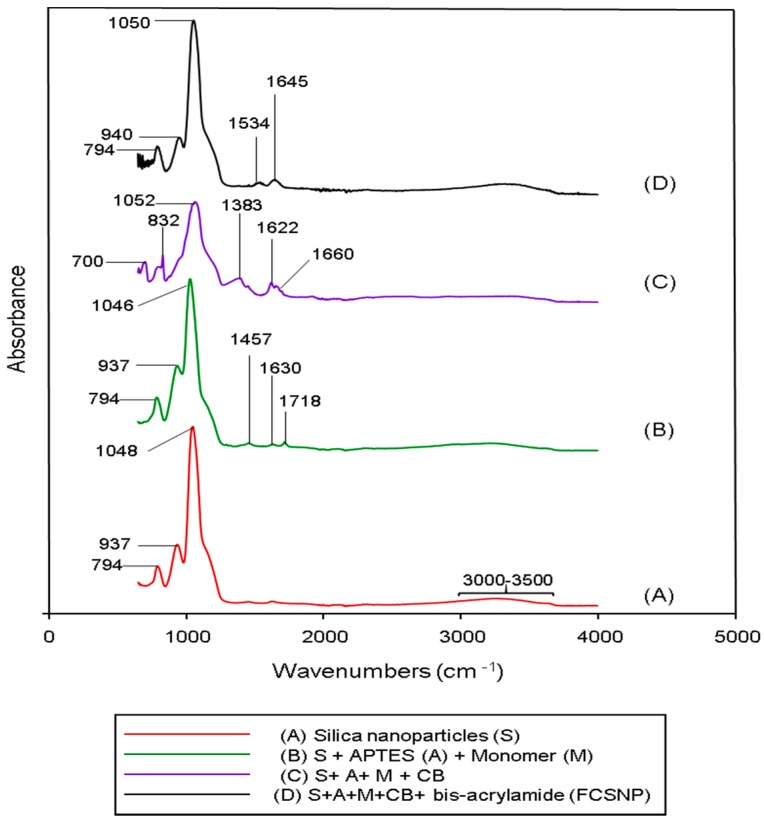
ATR-FTIR of the powders prepared from the nanoparticles at every stage of synthesis. (**A**) Corresponds to pure silica nanoparticles “S”; (**B**) is S + APTES “A” + monomer “M”; (**C**) is S + A + M + Cibacron blue “CB”; and (**D**) is S + A + M + CB + bis-acrylamide (FCSNP).

Further characterization included an Attenuated Total Reflection-Fourier Transformed Infrared (ATR-FTIR) analysis. Figure 2 shows the ATR-FTIR spectra of the silica nanoparticles and its consecutive modifications. In Figure 2A, four significant peaks for the silica nanoparticles are shown. One peak at 1048 cm^−1^ is due to the asymmetric vibration of Si-O-Si stretching bands, and another peak at 794 cm^−1^ corresponds to Si-O-Si symmetric vibration [23,24]. The next peak at 937 cm^−1^ corresponds to Si-OH and the last absorption peak around 3300 cm^−1^ is due to free O-H stretching bands [23]. Some of these absorption peaks were also observed in the following modifications. In Figure 2B, silica nanoparticles + APTES + monomer, new peaks appeared compared to the silica nanoparticle spectra. The 1630 cm^−1^ and 1457 cm^−1^ [25] related peaks are assigned to the NH_2_ deformation modes of the amine groups, while the 1718 cm^−1^ absorption peak corresponds to the C=O of the monomer [26].

Figure 2C shows the IR spectra of the nanoparticles after the addition of Cibacron blue. The absorption peaks at 1622 cm^−1^ and 1660 cm^−1^ are attributed to the vibration of N-H scissors [26] and the peak at 1383 cm^−1^ is due to the C=N stretching vibration in CB [26]. Finally, in Figure 2D, after the addition of bis-acrylamide, two new absorption peaks at 1645 cm^−1^ and 1534 cm^−1^ appeared. The first peak corresponds to Amide I and represents the -C=O stretching vibration of the amide group [27]. The second peak corresponds to Amide II, which is due to the -NH_2_ bending vibrations of the amine group [27,28]. These signals indicate the successful modification of the nanoparticles at every stage of synthesis.

**Figure 3:**
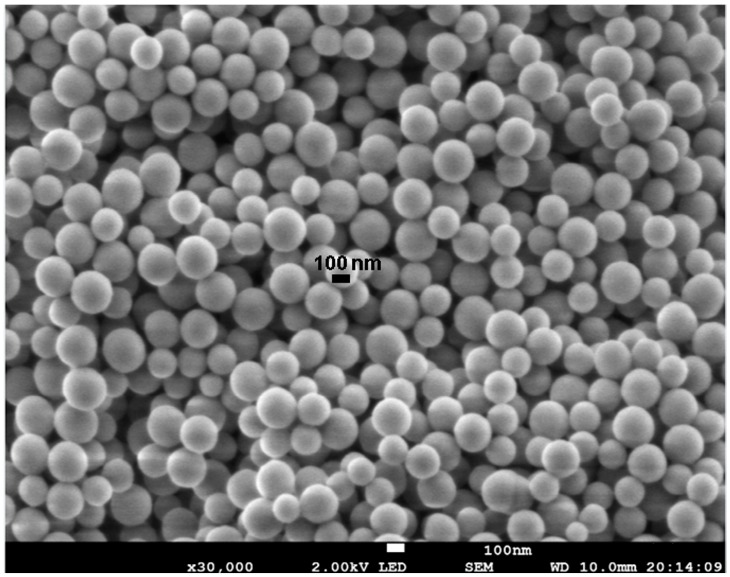
Morphological characterization of the first stage of synthesis of silica nanoparticles by scattering electron microscopy. Nanoparticles are spherical, with a diameter of ~140 nm, and are homogeneous.

The morphological characterization of the first stage of synthesis performed by SEM (Figure 3), showed a homogeneous distribution and the spherical shape of nanoparticles with a size of ~140 nm. This result was consistent with the size determined by dynamic light scattering (138.9 ± 5.6 nm).

**Figure 4:**
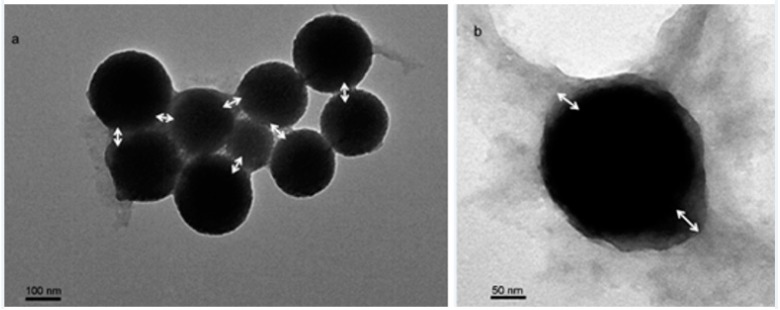
Morphological characterization of FCSNP by transmission electron microscopy. Particles are spherical, with a diameter of ~250 nm, and a polymeric shell is formed at the surface (white arrows). (**a**) and (**b**) were obtained at 3 K and 30 K magnifications, respectively.

The last stage of synthesis of FCSNP was characterized by TEM. Spherical nanoparticles of ~250 nm with a polymeric shell formed at the surface were observed (Figure 4). Size was consistent with dynamic light scattering (243.9 ± 11.6 nm) data. The polymer layer thickness with an average of 38 nm was estimated from Figure 4b.

### 2.2. Evaluation of the FCSNP in the Capture of Peptides

Using UHPLC-Q-ToF and a mixture of two model peptides, the interaction with Cibacron blue present at the FCSNP was tested (Table 2). The model peptides included a nonapeptide containing no hydrophobic region and alpha zein 34-mer with a long hydrophobic section. The nonapeptide was only obtained in the wash; this indicated that it was not captured by the molecular bait immobilized in the core of the nanoparticles. On the other hand, alpha zein34-mer was captured by the Cibacron blue [12,19], and eluted with all three types of eluents, indicating that the peptide-FCSNP interactions were of varying hydrophobicity. The presence of alpha zein 34-mer in the wash was due to the excess of the amount of peptide incubated with the FCSNP. The peptides were found according to their characteristic mono or multi-charged ions, which were [M + H] and [M + 2H] for the nonapeptide and [M + 3H] for alpha zein34-mer, respectively. The experimental molecular mass of the peptides from washes and elutions coincided with their respective theoretical mass.

In the nonapeptide sequence, there are only three hydrophobic amino acids, proline, isoleucine, and valine (Table 2, bold-faced print), and the hydrophobic interactions [12,19] were not sufficiently strong to bind the molecular bait in the FCSNP. On the other hand, in the amino acidic sequence of the peptide alpha zein 34-mer, 20 of 34 amino acid residues were hydrophobic (Table 2, bold-faced print), allowing strong, but varying, interactions with the molecular bait. Cibacron Blue is a pseudo-affinity molecular bait; therefore, it does not interact with proteins in a specific manner. However, it may interact with proteins with sufficiently long hydrophobic regions [19] regardless of their mass.

### 2.3. Effectiveness of Molecular Bait to Capture LMW Proteins

**Figure 5:**
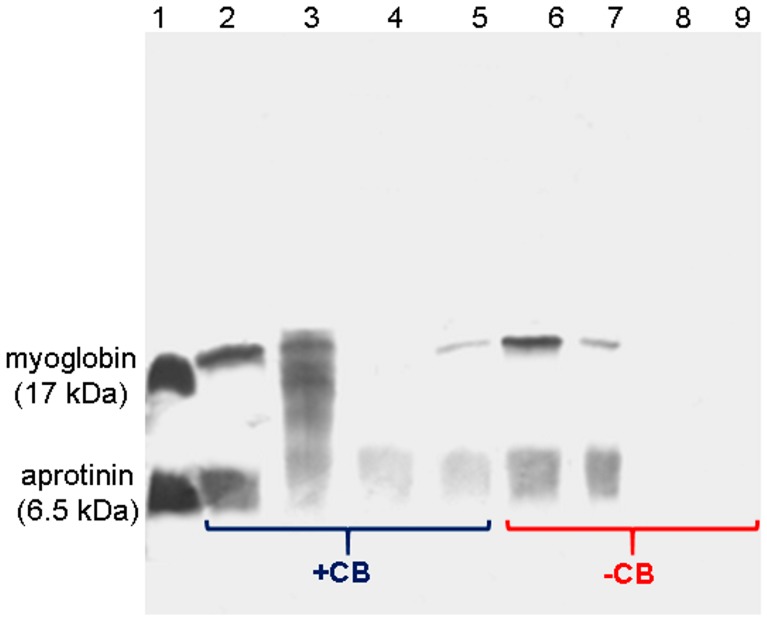
SDS-PAGE analysis of model proteins captured by FCSNP with (+CB) and without (−CB) Cibacron Blue. Model proteins, myoglobin, 17 kDa, and aprotinin, 6.5 kDa (lane 1); wash with PB (lanes 2 and 6); elution with 50% isopropanol (3 and 7); elution with 50% methanol (lanes 4 and 8), elution with ACN + NH_4_OH (lanes 5 and 9). Model proteins were obtained only in the elutions with 50% methanol and ACN + NH_4_OH with the +CB nanoparticles, and were absent from similar elutions with the −CB nanoparticles.

Myoglobin and aprotinin were incubated with FCSNP ± Cibacron Blue (+CB/−CB); washes and elutions were analyzed by SDS-PAGE (Figure 5). Washes with PB removed the excess of proteins (lanes 2 and 6). Proteins eluted with 50% (*v*/*v*) isopropanol are likely due to proteins in excess (lanes 3 and 7). The electrophoretic analysis showed that only the FCSNP containing CB allowed the capture of the model proteins due to the presence of their respective bands in elutions with 50% (*v*/*v*) methanol and ACN + NH_4_OH (lanes 4 and 5). On the contrary, nanoparticles synthesized without Cibacron Blue did not show an interaction with these proteins (lanes 8 and 9).

### 2.4. FCSNP in the Exclusion of HMW Proteins and Capture of LMW Proteins

**Figure 6:**
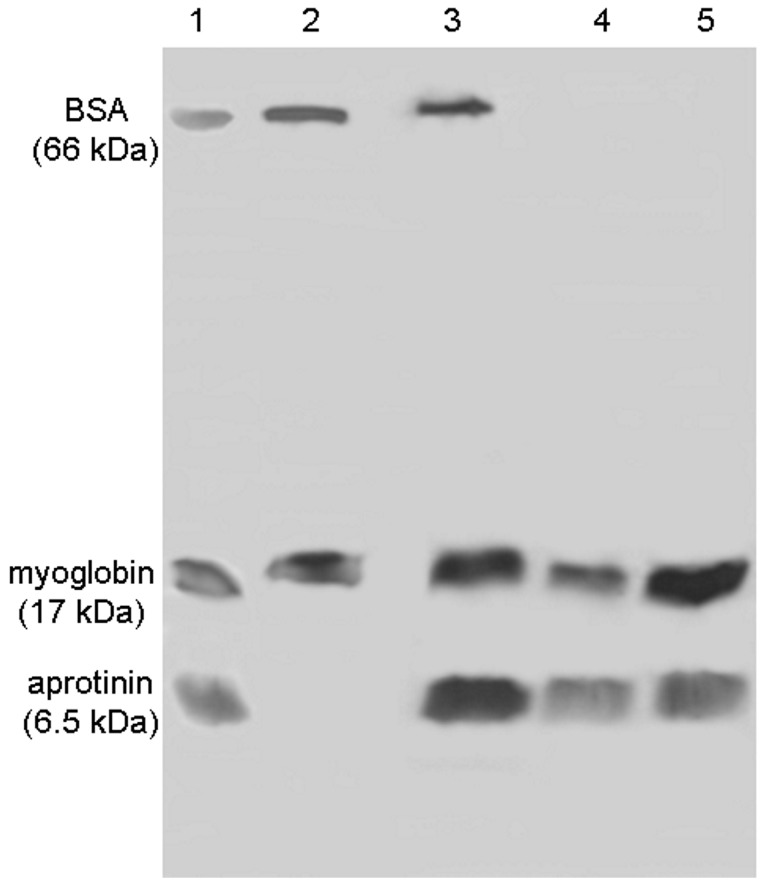
SDS-PAGE analysis of the FCSNP washes and elutions after incubation with high and low molecular weight model proteins for 60 min. Model proteins BSA (66 kDa), myoglobin (17 kDa), and aprotinin (6.5 kDa) (lane 1). Excess of proteins was removed in wash with PB (lane 2) and elution with 50% isopropanol (lane 3). Simultaneous exclusion of albumin and capture of myoglobin and aprotinin was achieved in elutions with 50% methanol and ACN + NH_4_OH (lanes 4 and 5, respectively).

The simultaneous exclusion of HMW proteins by the polymeric shell and capture of LMW proteins by molecular bait (bifunctionality) by FCSNP was analyzed by SDS-PAGE. Nanoparticles were incubated with a reduced amount of protein (ten-fold reduction compared to the previous experiment), while maintaining the ratio (3.5 µg of each protein, BSA, myoglobin and aprotinin) for 60 min (Figure 6). An excess of proteins were removed in the wash with PB (lane 2). Elution with 50% isopropanol obtained bands corresponding to BSA, as well as myoglobin and aprotinin (lane 3); this could indicate that these proteins were in excess or were interacting with molecular bait near the FCSNP surface. Thus, this elution could be considered as an intermediate step between washes and elutions.

Bifunctionality is evident in the elutions with 50% (*v*/*v*) methanol and ACN + NH_4_OH (lanes 4 and 5, respectively). Therefore, in order to obtain an optimal performance of the FCSNP, a lower amount of protein should be incubated to effectively remove HMW proteins present in the mixture and capture LMW proteins with a strong hydrophobic character.

Our results show the simultaneous exclusion of an HMW protein and capture of LMW proteins and peptides and coincide with those reported by Tamburro et al. [12]. However, FCSNP showed a size three times smaller (243.9 ± 11.6 nm) than the particles reported by Tamburro et al. (700 nm). In addition, silica is a highly stable compound with low reactivity with biomolecules, and it can be easily functionalized with diverse compounds including molecular baits offering great potential for a variety of applications [29]. Further, spherical shapes and narrow size distributions of silica nanoparticles can easily be reproduced [14]. FCSNP also remained stable after twelve months of storage (data not shown).

### 2.5. Estimation of the Nanoparticles Capacity

The capacity of the nanoparticles is expressed as the amount of protein captured per one milligram of nanoparticles (µg/mg). The results of the FCSNP capacity are shown in Table 3. The FCSNP capacity (3.1 ± 0.26–3.4 ± 0.15 µg/mg) is higher than that reported by Liu et al. [30], which captured 0.0054 µg/mg from an endoplasmic reticulum extract from a pig liver. However, these authors used molecularly imprinted nanoparticles that allowed the capture of proteins in a specific manner, while we used a pseudo-affinity molecule that allowed the capture of a variety of proteins. Thus, we might expect that the binding capacity of the FCSNP will vary depending on the proteins in the sample, and more specifically on the pivotal role of the amino acid sequence as the more hydrophobic regions within a protein structure, the stronger the interaction with the molecular bait [12,19]. These results suggest that FCSNP can serve to isolate biomarkers from complex biological fluids [1,2,3,12,31,32,33].

## 3. Materials and Methods

### 3.1. General Information

Cibacron Blue was purchased from Polysciences, Inc. (Warrington, PA, USA). Tetraethyl orthosilicate (TEOS), 3-aminopropyl triethoxysilane (APTES), 3-(Trimethoxysilyl) propyl methacrylate (monomer), *N*,*N*′-methylene-bis-acrylamide, ammonium persulfate, methanol, and isopropanol were from Sigma-Aldrich (St. Louis, MO, USA). Ethanol was acquired from Merck (Darmstadt, Germany). Ammonium hydroxide was supplied by VWR (Media, PA, USA). All other reagents were analytical grade. Nonapeptide was donated by Dr. M. de la Torre and alpha zein 34-mer by Dr. A.M. Calderon.

### 3.2. Synthesis of Functionalized Core-Shell Silica Nanoparticles (FCSNP)

The synthesis was carried out in four stages. Stage 1—silica nanoparticles were synthesized according to the Stöber method [17] with slight modifications. Briefly, a solution containing 40 mL of ethanol, 3 mL of ammonium hydroxide (final concentration of 1.81 M), and tetraethyl orthosilicate (final concentration of 77 mM) was made with continuous stirring at room temperature (RT) for 24 h (solution turned from transparent to white). Stage 2—to the previous solution were added; APTES (final concentration of 2.4 mM) and the monomer 3-(Trimethoxysilyl) propyl methacrylate (final concentration of 4.8 mM), the solution was incubated with continuous stirring at RT for 24 h. Once the core with chemical groups was obtained, the cores were washed with 80% (*v*/*v*) ethanol and centrifuged (2422× *g* for 12 min). An additional wash with carbonate buffer (50 mM, pH 9.5) was performed, supernatants were discarded, and the particle pellet was suspended in 30 mL of carbonate buffer.

Stage 3—Cibacron Blue was added to the solution (final concentration of 4 mM) and left at stirring at 40 °C for 24 h. After incubation, the particles were washed with ethanol and carbonate buffer and the particle pellet was re-suspended in carbonate buffer as described above. The solution was purged with nitrogen for 1.5 h. Stage 4—*N*,*N*′-methylene-bis-acrylamide and ammonium persulfate were dissolved in 20 mL of Milli-Q water, then added to the previous solution (final concentration of 6 mM and 8 mM, respectively). The reaction was maintained at RT under nitrogen for 6 h. Particles were washed once with Milli-Q water (2422× *g* for 12 min), the supernatant was discarded, and the resultant pellet was suspended in 20 mL of 20% ethanol. Aliquots of 1 mL were transferred to vials, dried by centrifugation in vacuum (Centrivap, Labconco, Kansas City, MO, USA) until a constant weight was obtained, and stored at 4 °C. As a control, synthesis was performed simultaneously without adding Cibacron Blue.

### 3.3. Size and Surface Charge Characterization of the Nanoparticles

The size and surface charge of nanoparticles were assessed by dynamic light scattering and zeta potential, respectively, using a nano-zetasizer (Nano-ZS 90, Malvern instrument, Malvern, UK). Measurements were performed at every stage of the process and recorded as the average of three test runs.

### 3.4. ATR-FTIR Spectroscopic Studies

A total of 750 µL of samples of every stage of synthesis were dried by centrifugation in vacuum and the corresponding powders were then analyzed. ATR-FTIR spectra were measured from 4000 to 650 cm^−1^ at a resolution of 4 cm^−1^ by a Cary 630 spectrometer (Agilent, Cary 630 FTIR Spectrometer, Santa Clara, CA, USA).

### 3.5. Morphological Characterization of the Nanoparticles

Nanoparticles from the first stage of synthesis were characterized by scattering electron microscopy (JEOL, JSM-7800F, SEM, Akishima, Tokyo, Japan) using an acceleration voltage of 2.0 kV and images were obtained with a magnification of 30,000×. Images of FCSNP were obtained by transmission electron microscopy (JEOL 1200 EX II TEM, Peabody, MA, USA) operating at 60 kV and images were obtained with 3 K and 30 K magnifications. In both cases (SEM and TEM experiments), the samples were air-dried before using them.

### 3.6. Evaluation of the FCSNP in the Capture of Peptides

One milligram of FCSNP was suspended in 100 µL of Milli-Q water and incubated with a 50 µL model peptides solution, for 60 min. This solution contained: 25 µL of alpha zein 34-mer, 3646.32 Da (United Biosystems, Herndon, VA, USA), and 25 µL of nonapeptide (nine amino acids, 965.90 Da; GenScript, Piscataway, NJ, USA) at a concentration of 1 µg/µL. After incubation, particles were washed three times with 50 mM phosphate buffer pH 7.7 (PB, 500 µL each) and eluted three times (each with 500 µL) with 50% (*v*/*v*) isopropanol, 50% (*v*/*v*) methanol, and then with 70% (*v*/*v*) acetonitrile + 20% (*v*/*v*) ammonium hydroxide (ACN + NH_4_OH). Following each wash and elution, particles were separated from the supernatant by centrifugation at 5585× *g* for 5 min. Respective supernatants were collected, combined in the same vial, and then dried by centrifugation in vacuum.

All samples were analyzed by ultra-high-performance liquid chromatography (UHPLC)-electrospray ionization-tandem mass spectrometry (MS) in positive mode. The chromatographic instrument was an Infinity 1290 (Agilent, Santa Clara, CA, USA) coupled to a quadrupole-time of flight (Q-TOF) mass spectrometer (Agilent 6530 Accurate-Mass Q-TOF, Santa Clara, CA, USA) with an ESI interface and automatic injector. The chromatographic column was a Zorbax 300 SB-C18 rapid resolution HD 2.1 mm × 50 mm with a 1.8 µm particle size (Agilent Technologies, Santa Clara, CA, USA) and the volume injected was 1 µL of each fraction (washes and elutions). The mobile phase flow rate was 250 µL/min and the column temperature was kept at 35 ± 0.8 °C.

A binary mobile phase with a gradient elution was used. Solvent A was 0.1% aqueous trifluoroacetic acid (TFA) and solvent B was 0.1% TFA in acetonitrile (ACN). The gradient was performed as follows: 70% of B increased to 90% in 5 min, decreased to 70% of B in 2 min, and was then constant for another 2 min. Separation by UHPLC was carried out in a 9 min run time. Optimized MS parameters were as follows: Nitrogen flow rate of 8 L/min, spray potential of 2000 V, nebulizer pressure of 50 psi, and source temperature of 300 °C.

### 3.7. Effectiveness of Molecular Bait in the Capture of LMW Proteins

One milligram of FCSNP was suspended in 100 µL of Milli-Q water and incubated for 60 min with a mix of model LMW proteins consisting of 35 µL of myoglobin, 17 kDa (GE Healthcare, Buckinghamshire, UK) and 35 µL of aprotinin, and 6.5 kDa (GE Healthcare, Buckinghamshire, UK) at a concentration of 1 µg/µL. After incubation, consecutive washes and elutions were performed as described above. Samples were suspended in Milli-Q water and the protein concentration was estimated by a Bradford assay [34]. The electrophoretic profile was obtained using 20% SDS-PAGE according to Laemmli [35] by loading 1 µg of protein from each fraction. Silver staining was performed according to Shevchenko et al. [36]. The entire procedure was performed simultaneously, using nanoparticles with and without Cibacron Blue (control).

### 3.8. FCSNP in the Exclusion of HMW Proteins and Capture of LMW Proteins

FCSNP were incubated with 3.5 µL of BSA (HMW protein), 3.5 µL of myoglobin, and 3.5 µL of aprotinin; each at a concentration of 1 µg/µL, for 60 min. The procedure was performed using FCSNP stored for twelve months at 4 °C. An analysis of proteins in the elutions was performed by 15% SDS-PAGE [35] by loading 1 µg of protein from each fraction. Polyacrylamide gels were stained [36].

### 3.9. Estimation of the Nanoparticles Capacity

Aprotinin or myoglobin (7 µg, 10 µg, 13 µg, and 16 µg) were dissolved in 100 µL of Milli-Q water and incubated separately with FCSNP (1 mg) for 60 min. After incubation, two washes with PB and two elutions with ACN + NH_4_OH were performed. The concentration of eluted proteins was determined by a Bradford assay [34].

## 4. Conclusions

In one step, the FCSNP managed to capture model proteins and peptides of a low molecular weight, while excluding high molecular weight proteins. Functionalization of nanoparticles with the molecular bait, Cibacron Blue, was crucial to capture the molecules of interest. The FCSNP were stable after twelve months of storage and demonstrated a capacity of 3.1–3.4 µg/mg.

## Figures and Tables

**Table 1:** Size and zeta potential of the silica nanoparticles at each stage of synthesis. Values are expressed as the mean ± standard deviation (SD) of three samples, each measured in triplicate.

	Stage of Synthesis			
	1	2	3	4
	Silica Nanoparticles	APTES and Monomer	Cibacron Blue	FCSNP
Size ± SD (nm)	138.9 ± 5.6	218.4 ± 4.7	211.5 ± 18.1	243.9 ± 11.6
Zeta potential ± SD (mV)	−47 ± 0.7	−34.2 ± 1.5	−54.6 ± 0.5	−38.1 ± 0.9

SD = Standard deviation; APTES = 3-aminopropyl triethoxysilane; Monomer = 3-(trimethoxysilyl) propyl methacrylate; FCSNP = Functionalized core-shell silica nanoparticles.

**Table 2:** Analysis by UHPLC-Q-ToF of wash and elutions from FCSNP after incubation with model peptides.

Peptide Sequence	Theoretical Mass (Da)	Experimental *m*/*z*		Error (Da)
		Wash* with 50 mM phosphate buffer	Consecutive elutions ** with 50% isopropanol, 50% Methanol and ACN + NH_4_OH ***	
1. YSSK**P**D**IV**G		965.49		−0.4352
	965.06	(M + H)^1+^		
			Not eluted	
		483.2519		0.5562
		(M + 2H)^2+^		
2. **L**QQ**AIAA**S				
N**IPL**S**PLLF**Q				
QS**PAL**S**LV**Q	3646.32	1216.3632	1216.3632	0.2304
S**LV**Q **T**IR		(M + 3H)^3+^	(M + 3H)^3+^	

1 = Nonapeptide; 2 = Alpha zein 34-mer; * = No interaction with Cibacron blue; ** = Interaction with Cibacron blue; *** = 70% acetonitrile + 20% ammonium hydroxide.

**Table 3:** Capacity of the nanoparticles. FCSNP were incubated with different model proteins. Values are expressed as mean ± standard deviation (SD) from measures by triplicate.

Model Protein	Capacity of FCSNP (µg/mg)
Aprotinin	3.4 ± 0.15
Myoglobin	3.1 ± 0.26

FCSNP = Functionalized core-shell silica nanoparticles; Capacity of FCSNP (µg/mg) = amount of protein (µg) captured per milligram of nanoparticles.
